# Contemporary Theory and Research

**Published:** 1893-06-24

**Authors:** 


### Contemporary Theory and Research.

### PRISON INFIRMARIES.

In an article in "L'Assistance," M. Rousselet calls attention to the unsatisfactory state of many of the prison infirmaries in Paris, affirming that the health service has always been and is still in a rudimentary state favourable to the development of epidemics in these establishments. Among other points which he selects for criticism is the diet prescribed for the unhappy infants who are born in the prison. Article 16 provides for rations for babies under five months half a litre of milk and 40 grammes of flour, to which liberal allowance half a litre of broth is added after the age of five months is attained. The doctor appears to have no power to order additions or alterations to the diet unless in the unlikely event of the infant surviving to the age of three years. It would certainly, as M. Bousselet argues, be more logical to render it possible for the doctor to regulate what nourishment the infant should take before that age. But why keep babies under prison rule at all? M. Bousselet also advocates the creation of a special department in each prison establishment for criminals who have developed some form of curable mania during their term of imprisonment.

### MONKEY LANGUAGE.

According to a letter which Professor Garner has written to his brother in regard to his experiments with the monkey language, he has succeeded beyond his wildest hopes. He has written down nearly 200 monkey words, and considers there are only 20 or 30 more which have escaped him at present. "Achru" means sun, fire, warmth, &c.; "kukcha" means water, rain, cold, and apparently anything disagreeable; "gosku" means food, the act of eating. Professor Garner travelled 120 miles N.E. of Sierra Leone, and established his battery with the phonograph and revolving mirror in a clump of banyans in a forest region swarming with monkeys. 'The glitter of the mirror soon attracted a host of chattering monkeys. I watched them for an hour, and then cautiously approached. They disappeared like magic when they saw me—all but one, a chimpanzee. When I got close to it I found that it took no notice of me, but stood as if transfixed, with widely opened eyes and dilated pupils, gazing at the mirror. There was a slight tremulous motion in the limbs and a spasmodic twitching of the ears. I could hardly believe it—the animal was hypnotised. It was making a guttural sound like 'achru.' When I subsequently listened to the phonogram I found that a similar sound was frequently recorded thereon amid what was then to me an unintelligible jumble of monkey chatter. I put the monkey in a bamboo cage, and on examining him about an hour afterwards found him still under the hypnotic influence. I revived him with a good strong sniff of ammonia, and held a lighted taper before his eyes. He was quite tractable and said 'achru,' and a few more tests satisfied me that this word embodied the idea of heat, light, warmth, and brightness. Other words followed, and it was wonderful to take note of his awakening intelligence."

### EXPERIMENTS ON LIVING ANIMALS.

At the instance of Mr. Herbert Gladstone, a Parliamentary return has been issued, showing the number of experiments performed under licence on living animals during the year 1892, distinguishing painless from painful experiments. The return for England and Scotland, which is signed by Dr. G. V. Poore, the inspector, states that the total number of licencees was 180, of whom 55 performed no experiments. There were 59 "licensed places" in 37 different situations. Four "licensed places" were added and five were removed from the registry in the course of the year. All licencees were restricted to the places specified on their licences with the exception of those who were permitted to perform inoculation experiments with the objects of studying outbreaks of diseases among animals in remote districts. The total number of the experiments performed was 3,916, of which 1,069 were physiological, 2,290 were pathological, and 601 therapeutical or pharmacological. Visits of inspection to the number of 122 were made to the various "licensed places." In Ireland, the return for which is signed by Mr. W. Thornley Stoker, two certificates expired and had not been renewed. No new licence was issued and one licence expired. Fourteen animals were experimented on, viz., thirteen rabbits and one dog. All experiments were done under anæsthetics, and were painless. Of these five were investigations of canine rabies, seven observations of the function of respiration, and two examinations of circulatory phenomena.

